# Prediction of posttraumatic functional recovery in middle-aged and older patients through dynamic ensemble selection modeling

**DOI:** 10.3389/fpubh.2023.1164820

**Published:** 2023-06-20

**Authors:** Nguyen Thanh Nhu, Jiunn-Horng Kang, Tian-Shin Yeh, Chia-Chieh Wu, Cheng-Yu Tsai, Krisna Piravej, Carlos Lam

**Affiliations:** ^1^International Ph.D. Program in Medicine, College of Medicine, Taipei Medical University, Taipei, Taiwan; ^2^Faculty of Medicine, Can Tho University of Medicine and Pharmacy, Can Tho, Vietnam; ^3^Department of Physical Medicine and Rehabilitation, School of Medicine, College of Medicine, Taipei Medical University, Taipei, Taiwan; ^4^Department of Physical Medicine and Rehabilitation, Taipei Medical University Hospital, Taipei, Taiwan; ^5^Graduate Institute of Nanomedicine and Medical Engineering, College of Biomedical Engineering, Taipei Medical University, Taipei, Taiwan; ^6^Professional Master Program in Artificial Intelligence in Medicine, College of Medicine, Taipei Medical University, Taipei, Taiwan; ^7^Department of Physical Medicine and Rehabilitation, Wan Fang Hospital, Taipei Medical University, Taipei, Taiwan; ^8^Department of Epidemiology and Nutrition, Harvard T. H. Chan School of Public Health, Harvard University, Boston, MA, United States; ^9^Nuffield Department of Population Health, University of Oxford, Oxford, United Kingdom; ^10^Emergency Department, Wan Fang Hospital, Taipei Medical University, Taipei, Taiwan; ^11^Department of Emergency, School of Medicine, College of Medicine, Taipei Medical University, Taipei, Taiwan; ^12^Centre for Transport Studies, Department of Civil and Environmental Engineering, Imperial College London, London, United Kingdom; ^13^Department of Rehabilitation Medicine, Faculty of Medicine, Chulalongkorn University, Bangkok, Thailand; ^14^Department of Chula Neuroscience Center, King Chulalongkorn Memorial Hospital, Bangkok, Thailand

**Keywords:** dynamic ensemble selection, machine learning, middle-aged patient, older patient, traumatic injury

## Abstract

**Introduction:**

Age-specific risk factors may delay posttraumatic functional recovery; complex interactions exist between these factors. In this study, we investigated the prediction ability of machine learning models for posttraumatic (6 months) functional recovery in middle-aged and older patients on the basis of their preexisting health conditions.

**Methods:**

Data obtained from injured patients aged ≥45 years were divided into training–validation (*n* = 368) and test (*n* = 159) data sets. The input features were the sociodemographic characteristics and baseline health conditions of the patients. The output feature was functional status 6 months after injury; this was assessed using the Barthel Index (BI). On the basis of their BI scores, the patients were categorized into functionally independent (BI >60) and functionally dependent (BI ≤60) groups. The permutation feature importance method was used for feature selection. Six algorithms were validated through cross-validation with hyperparameter optimization. The algorithms exhibiting satisfactory performance were subjected to bagging to construct stacking, voting, and dynamic ensemble selection models. The best model was evaluated on the test data set. Partial dependence (PD) and individual conditional expectation (ICE) plots were created.

**Results:**

In total, nineteen of twenty-seven features were selected. Logistic regression, linear discrimination analysis, and Gaussian Naive Bayes algorithms exhibited satisfactory performances and were, therefore, used to construct ensemble models. The k-Nearest Oracle Elimination model outperformed the other models when evaluated on the training–validation data set (sensitivity: 0.732, 95% CI: 0.702–0.761; specificity: 0.813, 95% CI: 0.805–0.822); it exhibited compatible performance on the test data set (sensitivity: 0.779, 95% CI: 0.559–0.950; specificity: 0.859, 95% CI: 0.799–0.912). The PD and ICE plots showed consistent patterns with practical tendencies.

**Conclusion:**

Preexisting health conditions can predict long-term functional outcomes in injured middle-aged and older patients, thus predicting prognosis and facilitating clinical decision-making.

## 1. Introduction

Traumatic injuries are a leading cause of morbidity and mortality in middle-aged and older individuals (1, 2). Due to various sociodemographic factors and frailty, these patients have posttraumatic complications, prolonged hospitalization, and a poor quality of life, which, in turn, increase the risk of functional disability (3–7). Although prognosis and clinical decision-making are highly complicated in injured middle-aged and older patients, these factors are crucial for ensuring optimal care and rehabilitation, which may minimize mortality rates and improve functional independence in these patients (8, 9).

Approximately 50% of injured older patients have comorbidities, which result in functional limitations and poor physical health (10). Severe complications are associated with certain demographic characteristics and preexisting diseases (e.g., diabetes, cardiovascular disease, liver disease, and psychological conditions) (11); these factors may delay functional recovery. In patients with hip bone fractures, neurological and renal disorders are associated with reduced performance of activities of daily living (ADL), as assessed using the Barthel Index (BI) (6). These findings imply that demographics and baseline health conditions are associated with the risk of functional dependence in injured middle-aged and older patients. Therefore, those features might be used to build the predictive model for predicting long-term functional outcomes in this clinical population, which could support physicians in clinical practice.

In medicine, machine learning (ML) is an effective approach for making diagnoses and predicting prognoses; ML models exhibit satisfactory performance on clinical data sets, which are generally highly dimensional and imbalanced (12). An ML model was successfully used to predict knee pain in middle-aged and older individuals by using demographic, body measurement, and blood test data; the sensitivity and specificity of the prediction were 0.72 and 0.71, respectively (13). Furthermore, the CatBoost algorithm was used to identify depression in this population (sensitivity: 0.71; specificity: 0.89) (14). A study using an ML model demonstrated that physiological biomarkers are associated with mortality, extremity mobility, and ADL in middle-aged and older individuals (15). In addition, previous studies also evaluated the predictions of ML models on poststroke functional recovery, supporting rehabilitation practice (16, 17). These findings suggest that ML explores complicated patterns in clinical data to predict functional outcomes in middle-aged and older individuals in different clinical conditions. However, to the best of our knowledge, no predictive ML model has been constructed to predict the risk of long-term functional dependence in this population on the basis of preexisting health conditions. Therefore, by adopting an ML approach, we investigated the prediction ability of ML models for the risk of functional dependence in middle-aged and older patients 6 months after injury on the basis of their demographic characteristics and preexisting health conditions. We hypothesized that baseline features can be used to classify patients with and without functional dependency 6 months after injury and to predict functional prognosis in clinical practice.

## 2. Patients and methods

### 2.1. Study design

The protocol of this prospective observational study was approved by the Institution Research Board of Taipei Medical University, Taiwan (approval number: N202002099). All participants provided informed consent. This study was conducted and reported following the STROBE checklist. The funders had no roles in analyzing the data, interpreting the data, or drawing study conclusions.

### 2.2. Participants and data sets

We included 670 middle-aged and older patients with primary injury who were admitted to the Emergency Department of Wan Fang Hospital, Taipei Medical University, Taiwan, between August 2020 and March 2022. The inclusion criteria were an age of ≥45 years and the ability to provide informed consent. After the completion of treatment, the researchers contacted eligible patients and explained the study to them. After informed consent was obtained, the patients were interviewed and under physical examination. Data regarding the patients’ sociodemographic characteristics, preexisting diseases, and baseline clinical characteristics were collected. For patients with severe injury, the interviews and assessments were conducted after their conditions had stabilized. Clinical events during hospitalization (e.g., intensive care unit [ICU] admission) were also recorded. After 6 months, a follow-up assessment was performed through telephonic interviews. All assessments were performed by experienced physicians and nurses. After excluding 9 patients who died in the hospital, 134 patients who were lost to follow-up, and 26 patients who died during follow-up, 527 patients were included in this study (Supplementary Figure S1).

Clinical characteristics were assessed using a standardized format. The BI, a 10-item scale that assesses ADL, was used to evaluate the patients’ baseline (preinjury) levels of functional dependence (18). The total BI score ranges from 0 to 100, and a BI score of ≤60 indicates severe or complete functional dependence (19). The Clinical Frailty Scale (CFS), a 9-point scale that assesses physical frailty and cognitive impairment, was used to evaluate overall frailty (20). The CFS score from one to three indicates that an individual is healthy or has well-controlled medical problems, whereas the CFS score from four to nine indicates that an individual has “very mild frailty” to “terminally ill” (20). The Charlson comorbidity index (CCI), a 17-item tool with high validity and reliability, was used to assess the risk of 1-year mortality in the patients (21). The death rate is smaller than 0.5% when the CCI score is equal to zero, whereas this rate is approximately 20–25% when the CCI score is higher than six (21). Furthermore, the revised trauma score (RTS) was used to assess functional outcomes after injury (22, 23). The injury severity score, which is used to assess the severity of injury in six body systems, was calculated for the patients upon admission to the emergency department (ED-ISS), during hospitalization (HOSP-ISS), and before discharge (ISS) (24). The RTS score smaller than seven might suggest the high rates of survival and the low rate of complication in an individual (22, 23).

### 2.3. Independent input and output features

We used the patients’ baseline health conditions to predict their functional recovery 6 months after injury. The initial input features were sociodemographic characteristics (e.g., age, sex, marital status, employment status, education level, and body mass index), causes of trauma, preexisting diseases (e.g., diabetes, hypertension, heart failure, chronic kidney disease, liver diseases, chronic obstructive pulmonary disorder, stroke, anemia, hip fracture, Parkinson’s disease, and dementia), clinical events (e.g., ICU admission and hospital rehabilitation), and baseline assessment scores (e.g., BI score, CCI score, CFS score, RTS, ED_ISS, HOSP_ISS, and ISS). The outcome feature was functional status determined using the patients’ BI scores calculated 6 months after injury; on the basis of their BI scores, the patients were categorized into two groups: functionally independent (BI >60) and functionally dependent (BI ≤60) groups (16, 25).

### 2.4. Descriptive statistical analysis

Data are presented as mean ± standard deviation values for continuous variables and number and percentage values for categorical variables. The functionally independent and dependent groups were compared in terms of demographic and clinical characteristics by using the independent samples *t* test (for continuous variables) or chi-square test (for categorical variables). The BI scores and the number of patients with functional dependency were compared between baseline and 6 months after injury by using paired *t* and McNemar tests, respectively. In addition, the validation and independent data sets were compared in terms of patient characteristics. A *p* value of <0.05 indicated statistical significance. R (version 4.1.2; R Foundation for Statistical Computing, Vienna, Austria) and Jeffrey’s Amazing Statistics Program (version 0.16.3; The JASP Team, 2020) were used for statistical analyses.

### 2.5. ML process

**Figure 1:**
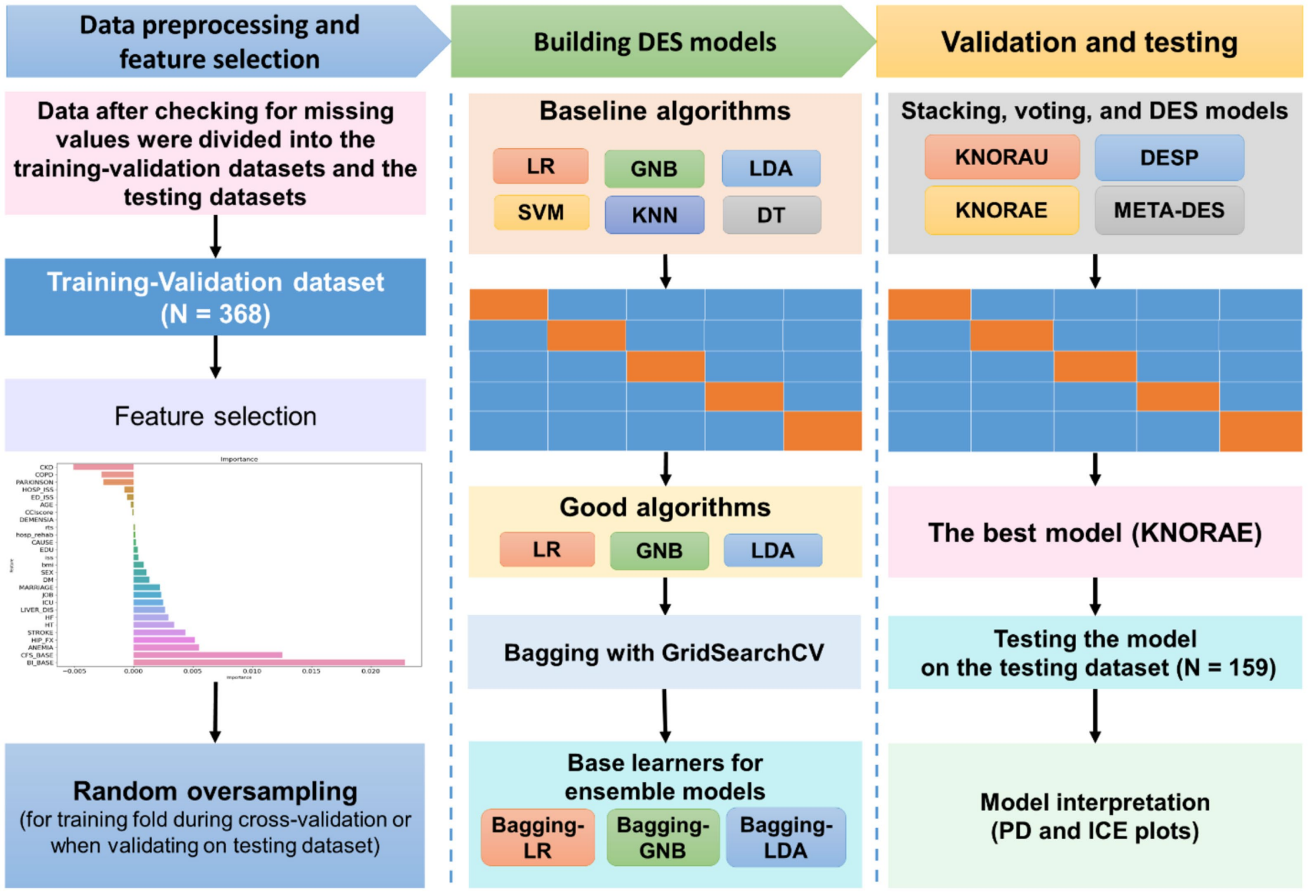
Machine learning process. Data obtained from the patients were first checked for missing values and outliners and then divided into training–validation data set (for model construction) and independent test (for model evaluation) data sets. The permutation feature importance method was used for feature selection. Because our data were imbalanced, random oversampling was applied on each training fold during cross-validation. Six algorithms, including support vector machine (SVM), logistic regression (LR), k-nearest neighbor (KNN), Gaussian Naive Bayes (GNB), linear discrimination analysis (LDA), and decision tree (DT), were validated through five-fold cross-validation. The algorithms exhibiting satisfactory performance (LR, GNB, and LDA) were subjected to bagging; afterward, they were used as the pool of classifiers for ensemble models (i.e., stacking, voting, and dynamic ensemble selection [DES] models). The ensemble models were compared in terms of performance, and the best model (k-nearest oracle elimination [KNORA-E]) was evaluated on the independent test data set. Partial dependence (PD) and individual conditional expectation (ICE) plots were constructed to investigate the interpretability of the model.

Figure 1 illustrates the ML process, which included data preprocessing, feature selection, model construction, model validation and testing, and model interpretation. All process steps were performed using Python 3.7 with Scikit-learn 1.1.3 (26) and DESlib library 0.3.5 (27).

#### 2.5.1. Data preprocessing

The data set was first analyzed for missing values and outliers and then randomly divided into training–validation and test data sets by the ratio 70:30 that is commonly used in ML (28, 29). The training–validation data set (*n* = 368) was used to train and construct ML models, whereas the independent test data set (*n* = 159) was used for evaluate the constructed models.

#### 2.5.2. Feature selection

The imputation feature importance method was used to select important features and exclude noise. The logistic regression (LR) algorithm was used as an estimator with 1,000 repeats. The features with negative or 0 important scores were eliminated. The remaining features were used to construct the ML model.

#### 2.5.3. Model construction

We first evaluated the classification performance of individual algorithms. The following six algorithms were selected: support vector machine (SVM), LR, k-nearest neighbor (KNN), Gaussian Naive Bayes (GNB), linear discrimination analysis (LDA), and decision tree (DT); these algorithms are commonly used for classification based on highly dimensional clinical data (13, 15, 16). Hyperparameter optimization was performed using GridSearchCV (Supplementary Table S1). The algorithms exhibiting poor performance were excluded.

For classification, an ensemble of classifiers is generally considered to be superior to a single classifier and could reduce the risk of overfitting (30). Therefore, the algorithms exhibiting satisfactory performance were subjected to bagging (a number of estimators were searched using GridSearchCV); then, these were used as base learners to construct stacking, voting, and dynamic ensemble selection (DES) models, including k-nearest oracle union (KNORA-U), k-nearest oracle elimination (KNORA-E), DES performance (DES-P), and meta learning for DES (META-DES).

#### 2.5.4. Cross-validation and internal validation on the test data set

The models were trained on the training–validation data set through stratified five-fold cross-validation, repeated twenty times. Because our data were imbalanced, random oversampling was separately applied on each training fold (but not the testing folds) during cross-validation. The DES and single-algorithm models were compared in terms of performance. The best model was evaluated on the independent test data set for assessing its performance on unseen data. In the cross-validation process, we estimated a 95% confidence interval for each performance indicator of all algorithms, defined by a mean score ± 1.96*validated standard error. When testing the final model on the independent test data set, the 95% CIs for performance indicators were calculated by conducting the bootstrap method (1,000 repeats) on the independent test data set.

#### 2.5.5. Performance matrix

The performance matrix included accuracy, sensitivity, specificity, F1 score, and area under the receiver operating characteristic curve (ROC-AUC). Because our aim was to investigate the prediction ability of the models for the risk of functional dependence, minimizing false negative rates was important. Therefore, we preferred sensitivity over specificity for model evaluation. In addition, because our data sets were imbalanced, a dummy classifier (uniform strategy) was used to construct a no-skill model, whose performance matrix was used as the baseline cutoff score for comparisons.

#### 2.5.6. Model interpretability analysis

Regarding the intuitive interpretation of the models, partial dependence (PD) and individual conditional expectation (ICE) plots were constructed to compare the effects of features on the outcomes predicted by the ML models with clinical tendencies. Given that the assessment scores indicated the effects of preexisting diseases on the model outcome, we constructed PD and ICE plots only for the assessment scores to evaluate the effects of changes in these scores on the risk of functional dependence 6 months after injury.

## 3. Results

### 3.1. Patient characteristics

Tables 1, 2 summarize the patient’s sociodemographic characteristics, injury severity levels, and functional outcomes. The most common cause of trauma was falling (69.1%; Table 1). Of the patients, >50% had at least one chronic disease. As shown in Table 2, the most common comorbidities were hypertension (53.7%), diabetes (29.2%), and heart failure (24.7%). The proportion of patients with functional dependency significantly increased 6 months after injury (from 5.6 to 11.4% in total; *p* < 0.01; Table 2). The patients’ demographic and clinical characteristics did not vary significantly between the training–validation and independent test data sets (*p* > 0.05; Tables 1, 2), which indicates that the independent test data set can be used for validation.

**Table 1:** Sociodemographic characteristics of the patients and causes of trauma.

Patient characteristics	Total (*N* = 527)	Training–validation data set (*N* = 368)	Independent test data set (*N* = 159)
Age (years), mean ± standard deviation	72.1 ± 12.8	72.4 ± 12.6	71.4 ± 13.1
Sex (women), *n* (%)	318 (60.3)	218 (59.2%)	100 (62.9)
Marital status, *n* (%)			
Single	32 (6.1)	25 (6.8)	7 (4.3)
Married	301 (57.1)	209 (56.8)	92 (57.9)
Divorced	34 (6.5)	23 (6.3)	11 (6.9)
Other	160 (30.3)	111 (30.1)	49 (30.9)
Job, *n* (%)			
Working	147 (27.9)	101 (27.5)	46 (28.9)
Housekeeping	27 (7.0)	28 (7.6)	9 (5.7)
Retired	329 (62.4)	230 (62.5)	99 (62.3)
Unemployed	14 (2.7)	9 (2.4)	5 (3.1)
Education level, *n* (%)			
None	51 (9.7)	35 (9.5)	16 (10.1)
Elementary	157 (29.8)	112 (30.4)	45 (28.3)
Secondary	59 (11.2)	47 (12.8)	12 (7.6)
High school	116 (22.0)	79 (21.5)	37 (23.3)
Undergraduate	125 (23.7)	82 (22.3)	43 (27.0)
Postgraduate	19 (3.6)	13 (3.5)	6 (3.7)
Cause, *n* (%)			
Fall	364 (69.1)	256 (69.6)	108 (67.9)
Traffic accident	133 (25.2)	90 (24.5)	43 (27.0)
Other	30 (5.7)	22 (5.9)	8 (5.1)

Patient characteristics did not vary significantly between the training–validation and independent test data sets (independent samples *t*-test or chi-square test).

**Table 2:** Clinical characteristics of the patients.

Preexisting health conditions	Total (*N* = 527)	Training–validation data set (*N* = 368)	Independent test data set (*N* = 159)
Health conditions, *n* (%)			
Hypertension	283 (53.7)	200 (54.4)	83 (52.2)
Diabetes	154 (29.2)	111 (30.2)	43 (27.4)
Heart failure	130 (24.7)	88 (23.9)	42 (26.4)
Anemia	84 (15.9)	64 (17.4)	20 (12.6)
Chronic kidney disease	66 (12.5)	48 (13.4)	18 (11.3)
Dementia	55 (10.4)	38 (10.3)	17 (10.7)
Stroke	49 (9.3%)	36 (9.8)	13 (8.2)
Hip bone fracture	31 (5.9)	25 (6.8)	6 (3.8)
Chronic obstructive pulmonary disorder	23 (4.4)	16 (4.4)	7 (4.4)
Parkinson’s disease	23 (4.4)	12 (3.3)	11 (6.9)
Liver diseases	20 (3.8)	13 (3.5)	7 (4.4)
Scores, mean ± standard deviation			
Baseline CFS	3.3 ± 1.5	3.3 ± 1.5	3.3 ± 1.5
Baseline ISS	7.1 ± 5.9	7.1 ± 5.9	7.2 ± 6.0
Baseline RTS	7.8 ± 0.2	7.8 ± 0.1	7.8 ± 0.2
BI			
Baseline	93.7 ± 15.16	93.9 ± 14.9	93.2 ± 15.7
After 6 months	86.8 ± 20.4***	86.6 ± 19.8***	87.2 ± 21.6***
Number of patients with functional dependency, *n* (%)			
Baseline	31 (5.9)	22 (6.0)	9 (5.6)
After 6 months	60 (11.4)**	42 (11.4)**	18 (11.3)**

Patient characteristics did not vary significantly between the training–validation and independent test data sets (using independent samples *t*-test or chi-square test). The BI scores and number of patients with functional dependency varied significantly between baseline and 6 months after injury when analyses were performed using the total, training–validation, and independent test data sets (paired t and McNemar tests, respectively). BI, Barthel Index; CFS, Clinical Frailty Scale; ISS, injury severity score; and RTS, revised traumatic score. **p < 0.01 and ***p < 0.001 compared with baseline.

### 3.2. Selection of informative features for classification

**Figure 2:**
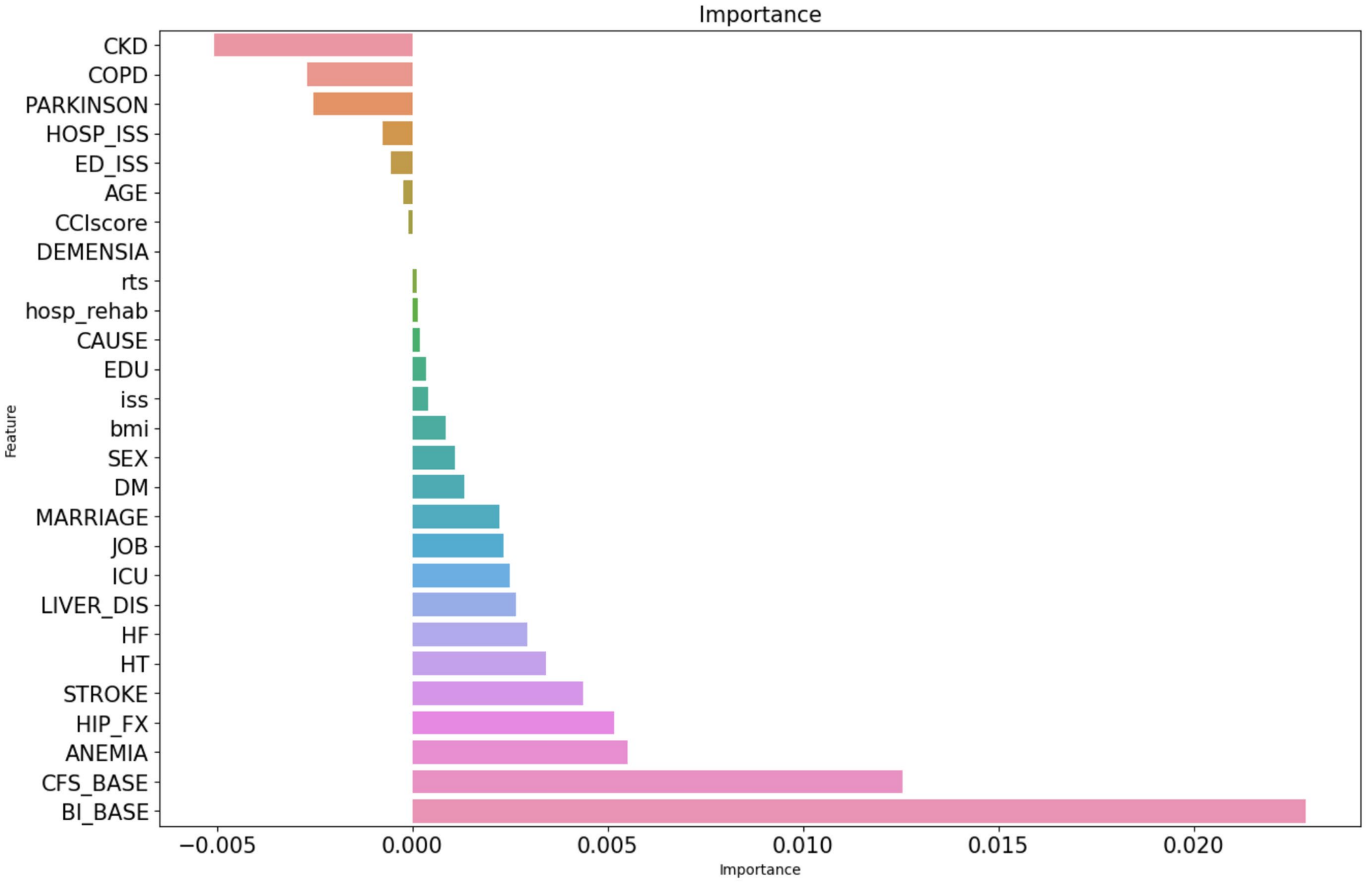
Permutation feature importance selection. The figure presents important scores of the features assessed using the permutation importance ranking method (1,000 repeats). CKD, chronic kidney disease; COPD, chronic obstructive pulmonary disorder; HOSP_ISS, injury severity score during hospitalization, ED_ISS, injury severity score upon admission to the emergency department; CCI score, Charlson comorbidity index score; RTS, revised traumatic score; EDU, education level; ISS, injury severity score before discharge; DM, diabetes mellitus; LIVER_DIS, liver diseases; HF, heart failure; HT, hypertension; HIP_FX, hip bone fracture; CFS_BASE, baseline score on the Clinical Frailty Scale; and BI_BASE, baseline score on the Barthel Index.

To reduce noise and select the most efficient features for predicting the risk of functional dependence 6 months after injury, feature selection was performed using the permutation feature importance method. Of the total 27 features, 8 had 0 or negative important scores and thus were removed (Figure 2). The remaining 19 features were used as input features to construct the classification models.

### 3.3. Performance of single-algorithm models for predicting the risk of functional dependence

Six single-algorithm classification models were constructed through hyperparameter optimization. The LR, LDA, and GNB models exhibited balanced performance. LR exhibited the highest sensitivity (0.789, 95% CI: 0.761–0.817) and specificity (0.789, 95% CI: 0.778–0.799), followed by GNB (sensitivity: 0.719, 95% CI: 0.690–0.745; specificity: 0.761, 95% CI: 0.739–0.783) and LDA (sensitivity: 0.687, 95% CI: 0.656–0.718; specificity: 0.777, 95% CI: 0.766–0.787). The F1 scores of the LR, LDA, and GNB models were higher than the baseline F1 score of the no-skill model, i.e., 0.462 (95% CI: 0.446–0.478), 0.416 (95% CI: 0.397–0.436), and 0.402 (95% CI: 0.386–0.419) vs. 0.181 (95% CI: 0.168–0.193), respectively. By contrast, the SVM, KNN, and DT models exhibited imbalanced performance with high specificity but low sensitivity (Table 3).

**Table 3:** Performance of single classifiers on the training–validation data set.

Classifiers	Accuracy	Sensitivity	Specificity	F1 score	ROC-AUC
SVM	0.900 (0.897; 0.903)	0.149 (0.124; 0.175)	0.997 (0.995; 0.998)	0.234 (0.197; 0.270)	0.632 (0.611; 0.652)
LR	0.789 (0.780; 0.798)	0.789 (0.761; 0.817)	0.789 (0.778; 0.799)	0.462 (0.446; 0.478)	0.854 (0.842; 0.866)
KNN	0.853 (0.846; 0.860)	0.381 (0.349; 0.413)	0.914 (0.907; 0.920)	0.367 (0.338; 0.393)	0.647 (0.631; 0.664)
GNB	0.756 (0.737; 0.775)	0.719 (0.690; 0.745)	0.761 (0.739; 0.783)	0.416 (0.397; 0.436)	0.811 (0.797; 0.826)
LDA	0.766 (0.757; 0.775)	0.687 (0.656; 0.718)	0.777 (0.766; 0.787)	0.402 (0.386; 0.419)	0.815 (0.801; 0.829)
DT	0.843 (0.835; 0.851)	0.328 (0.294; 0.362)	0.910 (0.902; 0.918)	0.317 (0.287; 0.347)	0.619 (0.602; 0.636)
No-skill model	0.500 (0.492; 0.507)	0.485 (0.452; 0.518)	0.501 (0.497; 0.505)	0.181 (0.168; 0.193)	0.500 (0.500; 0.500)

A no-skill model was constructed using a dummy classifier; the performance matrix of this model was used as the baseline threshold for comparison. SVM, support vector machine; LR, logistic regression; KNN, k-nearest neighbor; GNB, Gaussian Naive Bayes; LDA, linear discrimination analysis; DT, decision tree; and ROC-AUC, area under the receiver operating characteristic curve.

### 3.4. Performance of ensemble models for predicting the risk of functional dependence

**Figure 3:**
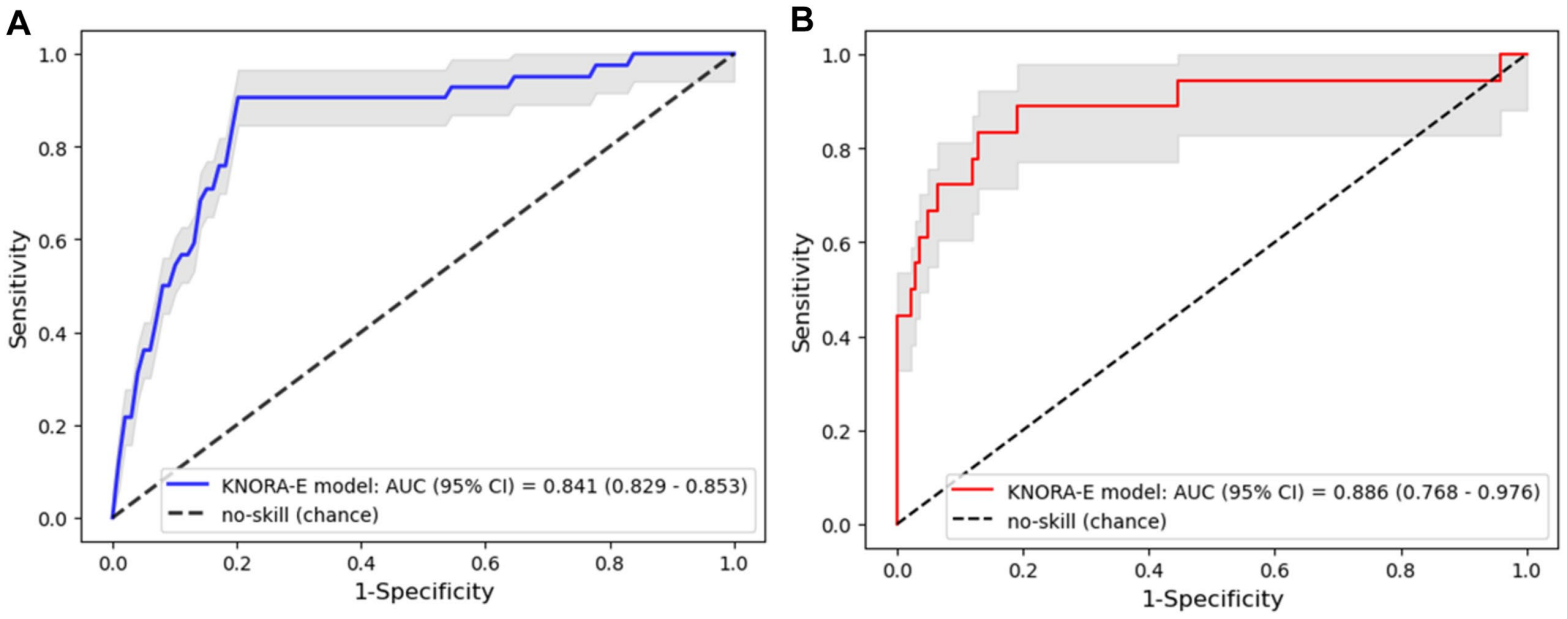
Area under the receiver operating characteristic curve (ROC-AUC) of the k-nearest oracle elimination (KNORA-E) model. **(A)** ROC-AUC of the KNORA-E model evaluated using the cross-validation data set. **(B)** ROC-AUC of the KNORA-E model evaluated using the test data set.

To investigate whether the ensemble models could improve the prediction ability of the input features, stacking, voting, and DES models were constructed through the bagging of LR, LDA, and GNB as the pool of classifiers (Table 4). The KNORA-E model outperformed the other ensemble models (sensitivity: 0.732, 95% CI: 0.702–0.761; specificity: 0.813, 95% CI: 0.805–0.822; Table 4; Figure 3A). Furthermore, the F1 score of this model (0.460, 95% CI: 0.444–0.477) was higher than the baseline F1 score of the no-skill model 0.181 (95% CI: 0.168–0.193) and the F1 scores of the other models.

**Table 4:** Performance of heterogeneous assemble models on the training–validation data set.

Classifiers	Accuracy	Sensitivity	Specificity	F1 score	ROC-AUC
KNORA-U	0.783 (0.775; 0.791)	0.719 (0.690; 0.748)	0.791 (0.782; 0.801)	0.431 (0.415; 0.446)	0.821 (0.808; 0.834)
KNORA-E	0.804 (0.796; 0.812)	0.732 (0.702; 0.761)	0.813 (0.805; 0.822)	0.460 (0.444; 0.477)	0.841 (0.829; 0.853)
DES-P	0.786 (0.777; 0.795)	0.713 (0.684; 0.741)	0.796 (0.786; 0.806)	0.431 (0.416; 0.451)	0.834 (0.823; 0.846)
META-DES	0.801 (0.793; 0.809)	0.668 (0.638; 0.697)	0.819 (0.810; 0.827)	0.434 (0.417; 0.452)	0.822 (0.809; 0.836)
Stacking	0.761 (0.752; 0.770)	0.707 (0.675; 0.739)	0.768 (0.758; 0.778)	0.404 (0.387; 0.421)	0.818 (0.804; 0.831)
Voting	0.780 (0.770; 0.790)	0.732 (0.702; 0.763)	0.786 (0.774; 0.798)	0.433 (0.417; 0.449)	0.836 (0.824; 0.848)
No-skill model	0.500 (0.492; 0.507)	0.485 (0.452; 0.518)	0.501 (0.497; 0.505)	0.181 (0.168; 0.193)	0.500 (0.500; 0.500)

A no-skill model was constructed using a dummy classifier; the performance matrix of this model was used as the baseline threshold for comparison. KNORA-U, k-nearest oracle union; KNORA-E, k-nearest oracle elimination; DES-P, dynamic ensemble selection performance; META-DES, meta learning for dynamic ensemble selection; and ROC-AUC, area under the receiver operating characteristic curve.

### 3.5. Validation on the independent test data set

To investigate the performance of the KNORA-E model on unseen data, the model was applied to the independent test data set. The performance matrix on the test data was similar to the performance on the train-validation data set, with accuracy, sensitivity, specificity, F1 score, and ROC-AUC values of 0.850 (95% CI: 0.792–0.899), 0.779 (95% CI: 0.559–0.950), 0.859 (95% CI: 0.799–0.912), 0.534 (95% CI: 0.359–0.680), and 0.886 (95% CI: 0.768–0.976), respectively (Figure 3B).

### 3.6. Model interpretability

**Figure 4:**
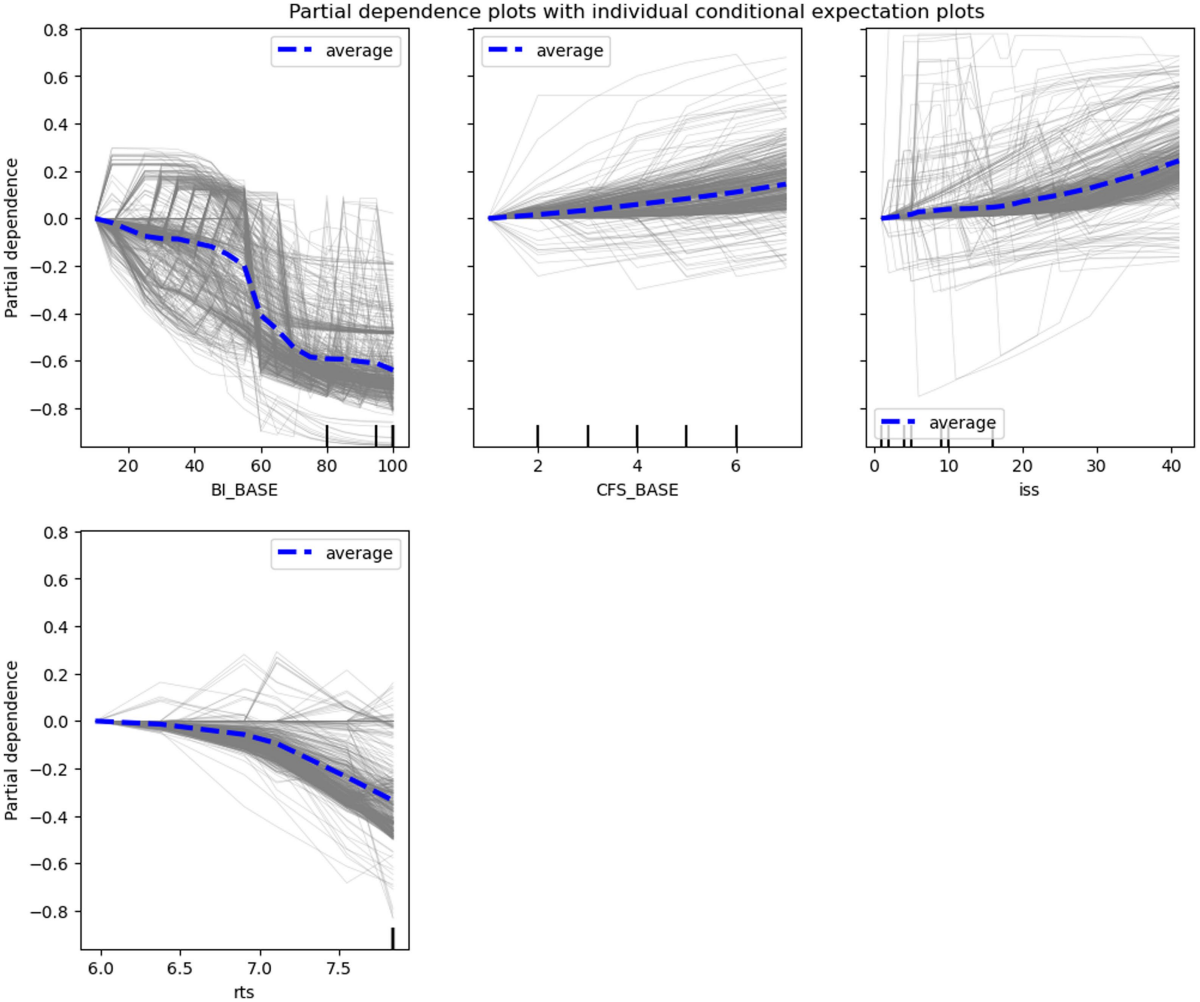
Partial dependence (PD) plots (blue lines) with individual conditional expectation (ICE) plots (grey lines). The plots indicate the overall (for PD) and individual (for ICE) effects of the features, including the baseline Barthel Index (BI_BASE) score, baseline Clinical Frailty Scale (CFS_BASE) score, revised traumatic score (RTS), and injury severity score (ISS).

PD and ICE plots were constructed for continuous predictive features (e.g., BI_BASE score, CFS_BASE score, ISS, and RTS) to investigate the effects of changes in the scores on the risk of functional dependence. These plots showed a consistent trend. The effects of predictive features on the model-predicted outcomes were consistent with the practical tendency. The risk of functional dependence decreased with increasing baseline BI scores and RTS, particularly when the scores were 40–60 and > 7, respectively. By contrast, this risk increased with increasing baseline CFS scores (nearly linear increase) and ISS scores, particularly when the score was >4 and > 20, respectively (Figure 4).

## 4. Discussion

Pathophysiological changes may worsen posttraumatic functional outcomes in middle-aged and older individuals (3–7). The pattern of functional recovery in these patients may vary from that in younger patients (2). Developing a model to predict long-term functional dependency in middle-aged and older individuals may facilitate treatment and rehabilitation, thus reducing the risk of functional disability. In the present study, using the DES models including selected features, we found that baseline sociodemographic characteristics, functional assessment scores, and preexisting diseases successfully predicted the risk of functional dependence in the study population 6 months after injury. The assessment scores may be non-linearly correlated with functional outcomes, and the correlation may be stronger in some score ranges. Our findings suggest that preexisting health conditions considerably affect functional recovery in injured middle-aged and older individuals; furthermore, ML models constructed using these input features can be used to predict prognosis in this population.

High dimensionality and imbalance are the characteristics of real-life clinical data; these characteristics complicate the analysis of data using ML models and reduce the applicability of these models (12). In the present study, the selection of features through the permutation importance method helped remove noise from the input features and improve model performance. Using the remaining features, several single-algorithm models, such as the LR, LDA, and GNB models, were constructed; these models predicted functional outcomes with acceptable performance. Although these algorithm exhibited acceptable classification performance on highly dimensional data after appropriate feature selection, they may exhibit overfitting in several circumstances, particularly in the case of data imbalance (31). Thus, we constructed heterogeneous ensemble models; these models may have a reduced overfitting risk and improved prediction ability (30). In the present study, the KNORA-E model exhibited the highest, balanced performance on both the training–validation and independent test data sets, which indicated that the levels of bias and variance were low for this model. Because of the nature of ML on extremely imbalanced data, the different gap between sensitivity and specificity in the final model in our study might be acceptable, which was similar to a previous study conducted an on extremely imbalanced cancer dataset (32). This finding corroborates that DES models exhibit improved classification performance on imbalanced data (33). Thus, DES modeling with feature selection may be an effective ML approach for prognosis using clinical data.

In this study, baseline BI and CFS scores (indicating functional level and frailty, respectively) were found to be the best features for predicting the risk of functional dependence in middle-aged and older patients 6 months after injury. The other assessment scores used in this study, such as RTS and ISS, also contributed to the model-based prediction of functional recovery. The BI is a common scale used for evaluating functional independence in patients with various health conditions; this scale has high validity and reliability (19). The CFS score indicates the requirement of additional health care support for injured patients (34). However, the BI and CFS scores are influenced by comorbidities, age, and other sociodemographic factors (18, 35). The use of only assessment scores may not be sufficient for effectively predicting long-term functional outcomes in injured patients; therefore, additional features, such as sociodemographic characteristics and preexisting health conditions, must be included in the models.

Several preexisting diseases, including anemia, hip bone fracture, hypertension, heart failure, stroke, liver disease, and diabetes, helped predict functional recovery in older patients 6 months after injury. Hypertension, stroke, and diabetes markedly reduced the function and quality of life in patients with those diseases, particularly middle-aged and older patients (36). Anemia reduces physical and cognitive functions, thus worsening functional outcomes in middle-aged and older individuals (37, 38). Hip fractures are common in this population; this reduces their mobility and quality of life (39–41). In a relevant study, approximately 40% of all patients with heart failure experienced moderate to severe difficulties in performing ADL; these challenges were associated with mortality and hospitalization (42). Liver diseases may alter the structure and function of the brain and heart, thus worsening patients’ functional outcomes (43, 44). In summary, preexisting diseases may worsen functional outcomes in injured middle-aged and older individuals and may help predict the risk of long-term functional dependence in this population.

Using PD and ICE plots, we found that several assessment scores nonlinearly affected the risk of functional dependence. Higher baseline BI (particularly >40) scores exerted stronger effects on functional outcomes, which is consistent with Sinoff’s interpretation that older individuals with BI scores of <40 may exhibit severe or complete functional dependence (45). In patients who experienced an acute stroke, those with BI scores of ≥40 exhibited considerable improvements in their ADL compared with those with BI scores of <40 (46). In the present study, CFS scores of >4 markedly increased the risk of functional dependence. According to the CFS guideline, patients with CFS scores of 4 exhibit limited performance of activities, and those with CFS scores of >5 require assistance in performing ADL (47). Prolonged hospitalization in acute medicine units has been reported in patients with CFS scores of >4 (48). In the present study, RTSs of >7 strongly reduced the risk of functional dependence; this is consistent with the findings of other studies indicating that an RTS of 7 serves as the cutoff value for predicting mortality and complication development in injured patients (22, 23). Taken together, the findings support the interpretability of our ML models and their feasibility in clinical practice.

This study has some limitations. First, our data sets were imbalanced; although we validated our models using the independent test data set, the patient sample was regional and may not represent the general population. Thus, external validation is necessary to evaluate the generalizability of the model. Second, we assessed functional outcomes only at baseline and the 6-month follow-up; thus, time-related changes in functional outcomes, which may differ across preexisting health conditions, could not be recorded. Finally, our model did not include preclinical data; thus, the predictive value of preclinical features for long-term functional outcomes could not be estimated.

In conclusion, our model constructed through feature selection and DES modeling exhibited high performance for predicting the risk of functional dependence in injured middle-aged and older patients on the basis of their sociodemographic characteristics and preexisting health conditions. The model showed practical interpretability. This study may facilitate further large-scale studies on the prediction ability of baseline information and its application for the prediction of long-term functional prognosis in injured patients.

## Data availability statement

The original contributions presented in the study are included in the article/supplementary material, further inquiries can be directed to the corresponding author.

## Ethics statement

The studies involving human participants were reviewed and approved by Taipei Medical University Joint Institution Research Board. The patients/participants provided their written informed consent to participate in this study.

## Author contributions

J-HK and CL: study design, manuscript revising, and editing. C-CW and CL: patient enrollment and data collection. NTN, J-HK, T-SY, C-CW, C-YT, KP, and CL: data analysis and interpretation. NTN, J-HK, and CL: manuscript drafting. All authors contributed to the article and approved the submitted version.

## Funding

This research was jointly supported by grants from the National Science and Technology Council (Grant number: MOST 109-2314-B-038-079), Wan Fang Hospital, Taipei Medical University (Grant number: 110-wf-f-5), National Taipei University of Technology and Wan Fang Hospital, Taipei Medical University Joint Research Program (Grant number: 112-wf-ntut-05), and Injury Prevention and Disaster Medicine Research Foundation. The funders had no role in the design of the study, collection or analysis of data, decision to publish, or preparation of the manuscript.

## Conflict of interest

The authors declare that the research was conducted in the absence of any commercial or financial relationships that could be construed as a potential conflict of interest.

## Publisher’s note

All claims expressed in this article are solely those of the authors and do not necessarily represent those of their affiliated organizations, or those of the publisher, the editors and the reviewers. Any product that may be evaluated in this article, or claim that may be made by its manufacturer, is not guaranteed or endorsed by the publisher.
