# Supplementary material for: Prediction of posttraumatic functional recovery in middle-aged and older patients through dynamic ensemble selection modeling
Source: Front Public Health. 2023 Jun 20;11:1164820. doi: 10.3389/fpubh.2023.1164820 (PMC10319009; doi:10.3389/fpubh.2023.1164820)
Supplement: Supplementary file 1 [file Table_1.DOCX]

**Table S1.** Optimized parameters used for each algorithm

| **Classifier** | **Parameters** |
| --- | --- |
| SVM | C = 0.1, gamma = ‘scale’, kernel = ‘sigmoid’ |
| LR | C = 0.1, penalty = ‘l1’, solver = ‘liblinear’ |
| KNN | algorithm = ‘auto’, n_neighbors = 1, p = 2, weights = ‘uniform’ |
| LDA | solver = ‘svd’ |
| GNB | var_smoothing = 1e-8 |
| DT | criterion = ‘entropy’, max_features = ‘sqrt’, splitter = ‘best’ |
| Bagging LR | Number of estimators = 50 |
| Bagging GNB | Number of estimators = 10 |
| Bagging LDA | Number of estimators = 10 |
| Stacking | Final_estimator = bagging_LR |
| DES models | k = 10 |

SVM, support vector machine; LR, logistic regression; KNN, k-nearest neigbor; LDA, linear discrimination analysis; GNB, Gaussian Naïve Bayes; DT, decision tree; DES, dynamic ensemble selection.
