# Supplementary figures and images for: Prediction of posttraumatic functional recovery in middle-aged and older patients through dynamic ensemble selection modeling
Source: Front Public Health. 2023 Jun 20;11:1164820. doi: 10.3389/fpubh.2023.1164820 (PMC10319009; doi:10.3389/fpubh.2023.1164820)

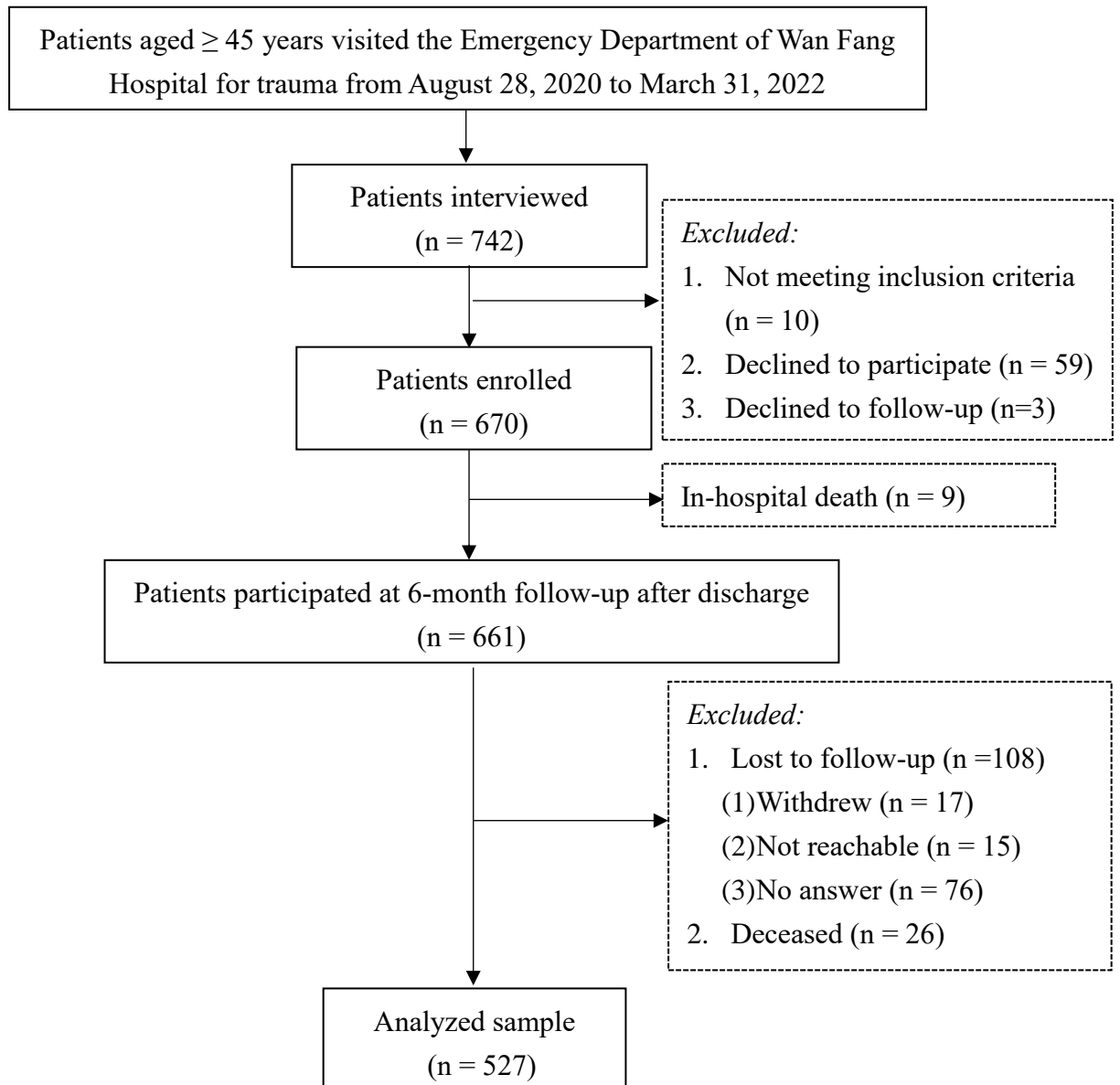

Supplement: Supplementary file 2 [file Data_Sheet_1.PDF]
